# Associations of Device‐Measured Physical Activity and Sedentary Time With Neural Responses to Visual Food Cues in Adults: A Functional Magnetic Resonance Imaging Study

**DOI:** 10.1002/hbm.70192

**Published:** 2025-03-12

**Authors:** Abdulrahman M. Dera, Elanor C. Hinton, Rachel L. Batterham, Melanie J. Davies, James A. King, Masashi Miyashita, Paul S. Morgan, Dimitris Papamargaritis, Julie Thompson, David J. Stensel, Alice E. Thackray

**Affiliations:** ^1^ National Centre for Sport and Exercise Medicine, School of Sport, Exercise and Health Sciences Loughborough University Loughborough UK; ^2^ College of Sport Sciences, Jeddah University Jeddah Saudi Arabia; ^3^ National Institute for Health and Care Research (NIHR) Bristol Biomedical Centre Diet and Physical Activity Theme, University of Bristol Bristol UK; ^4^ Oxford Medical Products Limited Witney UK; ^5^ Department of Medicine Centre for Obesity Research, University College London London UK; ^6^ NIHR University College London Hospitals Biomedical Research Centre London UK; ^7^ Diabetes Research Centre, University of Leicester Leicester UK; ^8^ NIHR Leicester Biomedical Research Centre, University Hospitals of Leicester NHS Trust and University of Leicester Leicester UK; ^9^ Faculty of Sport Sciences Waseda University Tokorozawa Japan; ^10^ Department of Sports Science and Physical Education The Chinese University of Hong Kong Hong Kong China; ^11^ Radiological Sciences School of Medicine, University of Nottingham Nottingham UK; ^12^ NIHR Nottingham Biomedical Research Centre Nottingham UK; ^13^ University Hospitals of Leicester NHS Trust, Infirmary Square Leicester UK

**Keywords:** appetite, brain, food cue reactivity, functional magnetic resonance imaging, physical activity, reward, sedentary behaviour

## Abstract

Self‐reported physical activity is associated with lower brain food cue responsiveness in reward‐related regions, but relationships utilizing objective physical activity measurement tools have not been explored. This cross‐sectional study examined whether device‐measured moderate‐to‐vigorous intensity physical activity and sedentary time are related to neural responses to visual food cues using functional magnetic resonance imaging. Fifty‐one healthy adults (30 men, 21 women; mean ± SD: age 26 ± 6 years; body mass index 24.1 ± 3.0 kg/m^2^) underwent a functional magnetic resonance imaging scan after an overnight fast while viewing images of high/very high‐energy density foods (HED), very low/low‐energy density foods (LED) and non‐food objects. Free‐living moderate‐to‐vigorous intensity physical activity and sedentary time were measured for seven consecutive days using an ActiGraph wGT3X‐BT and activPAL4 accelerometer, respectively. Associations of behavioural variables with brain food cue reactivity were examined in regression models controlling for physiological and behavioural covariates. After adjusting for age, sex, body mass index and device weartime, moderate‐to‐vigorous intensity physical activity was negatively associated with reactivity to LED versus non‐food cues in the precentral gyrus, hippocampus, posterior insula, and amygdala, which may diminish inhibitory‐related responses towards healthier lower energy value foods. Time spent in moderate‐to‐vigorous intensity physical activity was positively associated with reactivity to LED versus non‐food cues in the dorsal striatum, a region implicated in food motivation. A positive association was identified between sedentary time and reactivity to HED versus non‐food cues in the dorsal division of the posterior cingulate gyrus that has been implicated in attention allocation. These findings suggest that moderate‐to‐vigorous intensity physical activity may enhance the appeal of and motivation to consume LED foods, whereas sedentary time may promote attention towards HED foods, highlighting the potential for engaging in greater physical activity and less sedentary time to positively influence the central (brain) appetite control system.

## Graphical abstract

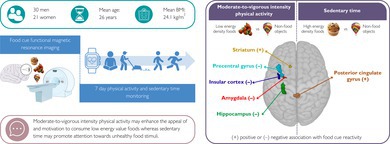

**Summary**

- We investigated associations of device‐measured moderate‐to‐vigorous intensity physical activity and sedentary time with neural responses to visual food cues using functional magnetic resonance imaging in men and women.
- Time spent in moderate‐to‐vigorous intensity physical activity was inversely related to reactivity to low‐energy value foods in the precentral gyrus, hippocampus, posterior insula, and amygdala, and positively associated with reactivity to low‐energy value foods in the dorsal striatum.
- Sedentary time was positively associated with reactivity to high‐energy value foods in the posterior cingulate gyrus.

## 1 Introduction

Research investigating the role of physical activity and sedentary time in appetite control has expanded in recent years due to the potential implications for weight management (Beaulieu et al. 2018). Key findings to emerge from this work are that individuals with higher levels of physical activity exhibit lower liking and wanting of high‐fat foods (Beaulieu et al. 2020), faster gastric emptying (Horner et al. 2015), and are better able to adjust energy intake after high‐energy preloads indicative of enhanced post‐meal satiety (Beaulieu et al. 2017). Furthermore, device‐measured moderate‐to‐vigorous intensity physical activity (MV‐PA) has been inversely related to disinhibition and binge eating behaviours, although these associations disappeared after adjustment for body fat, and opposing relationships were not apparent for sedentary behaviour (Myers et al. 2017). Nevertheless, the consensus of evidence suggests that regular physical activity has an overall positive influence on aspects of food reward and can enhance the sensitivity of the appetite control system (Beaulieu et al. 2020; Blundell and Beaulieu 2023).

The brain plays a critical role in human eating behaviour by integrating information on energy status from various hormonal, metabolic and chemical signals derived from peripheral organ and tissue sites (MacLean et al. 2017). In addition to monitoring nutritional requirements principally in the hypothalamus (Hussain and Bloom 2013), a diverse network of hedonic systems in the brain has a marked influence on eating behaviour and food intake (Berthoud et al. 2017). Data from blood‐oxygen‐level‐dependent (BOLD) functional magnetic resonance imaging (fMRI) studies have advanced understanding of the brain regions implicated in food reward and motivation including the hippocampus, insula, orbitofrontal cortex (OFC) and striatum, which are often identified during food cue reactivity paradigms (Huerta et al. 2014; Tang et al. 2012; van der Laan et al. 2011; van Meer et al. 2015). Many of these regions are also receptive to metabolic inputs from the body supported by evidence that the orexigenic gut hormone ghrelin increases neural responses to food cues in regions such as the amygdala, OFC, anterior insula and striatum (Malik et al. 2008; Goldstone et al. 2014). In contrast, the satiety gut hormones glucagon‐like peptide‐1 (GLP‐1) and peptide tyrosine tyrosine (PYY) have been shown to exert opposing anorexigenic effects on appetite‐related brain circuits (de Silva et al. 2011).

Evidence indicates that higher levels of physical activity can positively influence how people interact with food (Loprinzi et al. 2014). Acute and chronic exercise has been shown to reduce brain food cue responsiveness, particularly to images of high‐calorie foods, in several regions involved in reward and motivation, such as the insula, hippocampus, OFC and putamen (Dera et al. 2023). Moreover, cross‐sectional evidence suggests that higher levels of physical activity are associated with lower reactivity to food cues in several brain regions, including the insula, OFC, postcentral gyrus and precuneus (Drummen et al. 2019; Killgore et al. 2013; Luo et al. 2018). Conversely, time spent engaged in sedentary behaviour has been associated with higher brain food cue responsivity in the mid‐insula and amygdala (Luo et al. 2018). However, this understanding has evolved from a limited evidence base in which physical activity and sedentary behaviour have been assessed using self‐report tools (e.g., questionnaires and interview recall) that are prone to overreporting (Dhurandhar et al. 2015). Technological advances have improved the ability to objectively measure physical activity and sedentary time in free‐living environments (Dhurandhar et al. 2015; Edwardson et al. 2017). However, the use of these tools has yet to be combined with fMRI assessments of food cue reactivity.

The primary aim of this study was to examine the association of device‐measured, free‐living MV‐PA and sedentary time with central (brain) responses to food images using fMRI. As a secondary aim, we investigated the association of appetite perceptions, appetite‐related hormones and glucose with neural responses to food cues. It was hypothesised that brain food cue responsiveness would be inversely associated with MV‐PA and positively associated with sedentary time in a sample of healthy men and women.

## 2 Methods

### 2.1 Ethical Approval and Participants

The ethics advisory committee of Loughborough University approved this research. Fifty‐one healthy men and women provided written informed consent to participate in the study. The participants ranged in age from 18 to 44 years, were non‐smokers, and had no known medical conditions (e.g., diabetes or heart disease) or clinically diagnosed eating disorders. Participants were not taking any medications that might affect appetite (e.g., antibiotics, selective serotonin reuptake inhibitors), were not dieting and were weight stable (< 3 kg change in body mass in the last 3 months). Furthermore, participants were eligible if they had no food allergies or strict dietary practices (e.g., nut allergy, lactose intolerance, vegetarian or vegan diet) and consumed a Western European or Mediterranean‐style diet to increase the likelihood that participants were familiar with and consumed the food items presented during the food cue paradigm. Participants were eligible to undertake a 30‐min fMRI scan, and female participants had a regular menstrual cycle (oral contraceptives were allowed).

### 2.2 Preliminary Assessments

During a preliminary screening visit, eligibility for participation was confirmed and participants completed questionnaires to assess general health status, MRI safety and eating behaviour traits (51‐item Three‐Factor Eating Questionnaire). The three factor eating questionnaire assesses three domains of eating behaviour: cognitive restraint (intentional control of food intake to influence body weight), disinhibition (loss of control over eating in response to emotional and social cues), and hunger (subjective feelings of hunger and food craving) (Stunkard and Messick 1985). A standard measuring device (Seca 285, Seca GmbH & Co. KG, Hamburg, Germany) was used to determine the participants' body mass and stature, and body mass index (BMI) was calculated. Waist circumference was measured at the narrowest part of the torso between the lower rib margin and iliac crest to provide an indirect assessment of central adiposity. Participants were then familiarised with the format of the food cue paradigm presented during the fMRI scan (described below) but were not shown the full task until the main trial to retain the novelty of the images.

### 2.3 Main Trial

Participants arrived at the laboratory between 08:00 and 10:00 after fasting overnight for at least 10 h to complete a 3‐h main trial. Prior to the main experimental trial, participants were asked to complete a 24 h weighed diet record. During the same period, participants abstained from alcohol, caffeine and strenuous physical activity. Participants were provided with a standardised meal to consume between 19:00 and 21:00 in the evening before the main trial. The meal consisted of a pizza (2577 kJ energy, 16% protein, 52% carbohydrate, 32% fat) that was easy for the participants to prepare in the home environment, could be served uniformly for all participants and was expected to yield high compliance. After this meal, participants were instructed to consume no other food or drink (except plain water) before arriving at the laboratory the next day.

After arrival at the laboratory for the main trial, body mass and body fat percentage were recorded using bioelectrical impedance analysis (Seca mBCA 515; GmbH & Co. KG, Hamburg, Germany). After 20 min of seated rest, a fasted venous blood sample was collected to measure appetite‐related hormone and glucose concentrations, and participants were asked to complete ratings of perceived appetite immediately before and after undertaking an fMRI scan involving a food cue paradigm. Before leaving the laboratory, participants were provided with accelerometers that monitored their physical activity and sedentary time for eight consecutive days.

### 2.4 Appetite Perceptions

Ratings of perceived appetite (hunger, satisfaction, fullness, prospective food consumption) were assessed using 100‐mm visual analogue scales (Flint et al. 2000). An overall appetite score was calculated after reverse scoring the ratings for satisfaction and fullness (hunger + [100 − satisfaction] + [100 − fullness] + prospective food consumption), and an average of the pre‐ and post‐fMRI scan ratings was used in the final analysis.

### 2.5 Biochemical Analysis

Blood samples were collected into pre‐chilled potassium ethylenediaminetetraacetic acid (K2EDTA) S‐Monovettes (Sarstedt, Nümbrecht, Germany) to determine plasma total GLP‐1, total PYY, acylated ghrelin and glucose concentrations. Monovettes for total GLP‐1, total PYY and glucose were centrifuged immediately at 2383 g for 10 min at 4°C (Burkard, Hertfordshire, UK) and the plasma supernatant was aliquoted and stored at −80°C. Monovettes for acylated ghrelin were pre‐treated with a 50 μL solution containing potassium phosphate buffer solution, p‐hydroxymercuribenzoic acid and sodium hydroxide, which prevented protease degradation of acylated ghrelin. After blood collection, pre‐treated monovettes were centrifuged immediately at 2383 g for 10 min at 4°C, and 200 μL of 1 M hydrochloric acid was added to 2 mL of the plasma supernatant in a separate storage tube before the samples were re‐centrifuged (2383 g for 10 min at 4°C) and aliquoted for storage at −80°C.

Commercially available enzyme‐linked immunosorbent assays were used to determine plasma concentrations of total GLP‐1 and total PYY (Sigma Aldrich Corporation, St. Louis, USA) and acylated ghrelin (Bertin Bioreagent, Montigny le Bretonneux, France). The concentration of plasma glucose was determined using enzymatic, colorimetric methods on a benchtop analyser (Pentra 400, HORIBA Medical, Montpellier, France). Within‐batch coefficients of variation were as follows: total GLP‐1 3.7%, total PYY 4.9%, acylated ghrelin 4.4% and glucose 0.3%.

### 2.6 Device‐Measured Physical Activity and Sedentary Time

Free living MV‐PA and sedentary time were measured over eight consecutive days using a wrist‐worn ActiGraph wGT3X‐BT (Pensacola, Florida USA) and a thigh‐worn activPAL4 (PAL Technologies Ltd., Glasgow, UK) monitor, respectively, worn continuously on the non‐dominant side apart from water‐based activities. Wrist accelerometry infers sedentary behaviour from a lack of movement (i.e., inactivity) which can misclassify upright activity with limited movement as sedentary, whereas the activPAL4 provides accurate classification of sitting/lying posture (Edwardson et al. 2017) so it was adopted to improve the sedentary time measurement. The first day was removed during data processing to reduce potential bias arising from awareness that behaviour was being monitored (Baumann et al. 2018). Participants were asked to complete a daily log whilst wearing the accelerometers to document their sleep and wake times, structured exercise sessions, and device removal. For inclusion in the analysis, a minimum of 4 days (including one weekend day) of valid data across the 7‐day measurement period was required, which could be different days for MV‐PA and sedentary time.

ActiGraph wGT3X‐BT devices were deployed at a frequency of 100 Hz, and raw data files were downloaded via ActiLife v6.13.4 (ActiGraph, Pensacola, Florida, USA) for subsequent analysis using the open‐source R package GGIR v2.9.0 (Migueles et al. 2019). Analysis included automatic calibration, identification of unusually high values, detection of non‐wear, and calculation of the average magnitude of dynamic acceleration corrected for gravity (Euclidean Norm minus 1 g [ENMO]) averaged over 5‐s epochs. Days containing < 16 h of data or a calibration error > 10 milli‐gravitational (mg) units were excluded. A raw acceleration threshold of < 44.8 mg and ≥ 100.6 mg was used to define inactivity and MV‐PA, respectively, calculated in average minutes per day (Hildebrand et al. 2014, 2017), and MV‐PA data were expressed in bouts ≥ 1 min to mitigate the inclusion of incidental arm motion.

The activPAL4 monitor was attached to the midline of the anterior thigh using a nitrile sleeve and hypoallergenic dressing. Monitors were initialised at a frequency of 20 Hz with a dynamic range of ±4 g, and raw data files were downloaded using the manufacturer's software (PAL Technologies Ltd., Glasgow, UK). Analysis was performed using the java‐based application Processing PAL v1.32 (Leicester, UK) which implements a validated algorithm to establish waking wear time for each valid day (Winkler et al. 2016). Heat maps of processed data were inspected alongside the daily log to correct any misclassified data. A valid waking day was defined as ≥ 10 h of valid waking data, < 95% of time spent in any one behaviour and ≥ 500 steps. Average daily time (minutes) spent sedentary (sitting or lying) was extracted for analysis.

### 2.7 Food Cue Paradigm

**FIGURE 1:**
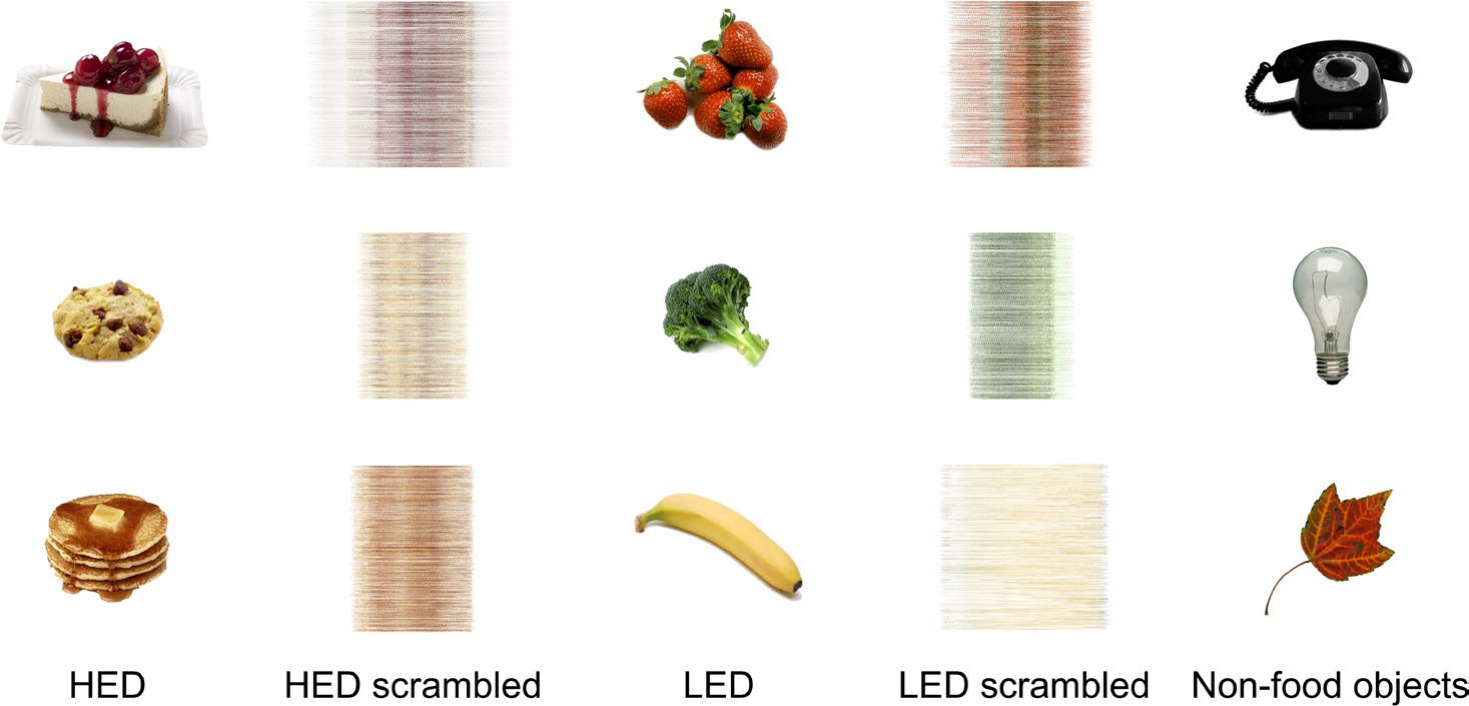
Example images presented during the food cue paradigm: High‐ and very high‐energy density food (HED), very low‐ and low‐energy density food (LED), non‐food objects and scrambled images of the two food categories. Images were acquired from a freely available image database (Blechert et al. 2014, 2019).

The fMRI food cue paradigm has been described previously (Thackray et al. 2023) and consisted of four categories of colour images acquired from an image database (Blechert et al. 2014, 2019) that were displayed in a block design: (1) high‐ and very high‐energy density foods (HED; *n* = 40); (2) very low‐ and low‐energy density foods (LED; *n* = 40); (3) non‐food objects (*n* = 40); and (4) scrambled images of the two food categories (*n* = 80). Scrambled images of the food items provided a low‐level baseline that did not identify the food but allowed the control of colour and visual characteristics (Goldstone et al. 2014). Examples of the images presented are shown in Figure 1. Food images were classified by energy density calculated using the energy value (kcal) and food weight (g) provided in the image database (Blechert et al. 2014, 2019), with a cut‐off of ≤ 1.5 kcal/g applied to classify LED foods (range 0.09–1.40 kcal/g) and ≥ 2.26 kcal/g for HED foods (3.11–7.41 kcal/g) (Vernarelli et al. 2018). The food and non‐food image categories were matched for visual characteristics, including colour, object size, brightness, contrast and complexity.

The paradigm included eight blocks consisting of five HED images, five LED images, and five non‐food object images, which were interleaved with 16 blocks of scrambled images. Images were displayed in a random order for 2.5 s, separated by a 0.5 s inter‐stimulus interval of a central fixation cross, resulting in a total duration of 10 min 10 s. The visual stimulus was displayed on an MRI‐compatible monitor using the software package Presentation version 21.1 (Neurobiobehavioural Systems Inc., California, USA). Images were slightly offset from the screen centre, and participants were asked to indicate left or right screen position using a hand‐held button box connected to a Lumina LSC‐400 controller (Cedrus Corporation, California, USA).

### 2.8 fMRI Data Acquisition

The MRI scans were acquired using a GE 3T Discovery 750w scanner (General Electric, Boston, USA) equipped with a 32‐channel head coil. A sagittal 3D fast spoiled gradient echo (FSPGR) structural scan was acquired with the following parameters: time to echo (TE) = 3.1 ms; flip angle = 8°; field of view = 240 mm; slice thickness = 1 mm; voxel size = 1 × 1 × 1 mm; scan time = 4 min 48 s. The food cue fMRI scans were performed in a single run using a non‐product simultaneous multi‐slice (SMS) echo‐planar imaging (EPI) sequence with the following parameters: TE = 35 ms; repetition time (TR) = 1900 ms; flip angle = 70°; field of view = 211 mm; SMS factor = 2; 46 slices; slice thickness = 2 mm; number of volumes = 340; voxel size = 2.2 × 2.2 × 2.2 mm; and scan time = 10 min 46 s. To correct for susceptibility‐induced distortions, two spin‐echo EPI images with reverse phase‐encoding directions (posterior > anterior and anterior > posterior) were also collected during each scan: TE = 41 ms; TR = 3800 ms; flip angle = 90°; field of view = 211 mm; 46 slices; slice thickness = 2 mm; and voxel size = 2.2 × 2.2 × 2.2 mm.

### 2.9 fMRI Data Analysis

The fMRI Expert Analysis Tool (FEAT) was used for the pre‐processing and analysis of fMRI data (Woolrich et al. 2001, 2004) in FMRIB's Software Library (FSL) version 6.0.4 (Smith et al. 2004). Raw DICOM images acquired from the MRI scanner were converted to NIfTI format using the dcm2niix tool (Li et al. 2016). The fsl_anat tool was used to remove non‐brain structures from the T1 structural images. Pre‐processing of the functional data involved motion correction using MCFLIRT (Jenkinson et al. 2002), spatial smoothing using a full‐width half‐maximum Gaussian kernel of 5 mm, global intensity normalisation, susceptibility‐induced distortion correction using top‐up (Andersson et al. 2003; Smith et al. 2004), removal of motion‐related artifacts with the tool ICA‐AROMA (Independent Component Analysis—Automatic Removal of Motion Artifacts) (Pruim et al. 2015) and a high‐pass filter cut‐off of 90 s. Functional image registration to structural and standard (MNI152_T1_2mm_brain) images was performed using FMRIB's Linear Image Registration Tool (FLIRT) (Jenkinson et al. 2002) and the Nonlinear Image Registration Tool (FNIRT) (Andersson et al. 2007).

### 2.10 Whole‐Brain Analysis

At the first level, explanatory variables were entered into a general linear model in FSL's FEAT for each image category (HED, LED and non‐food cues) with a separate model performed for each participant. To examine responses to food cues minus non‐food objects, three contrasts were defined: (i) food [HED + LED] > non‐food cues; (ii) HED > non‐food cues; and (iii) LED > non‐food cues. To account for saturation effects, the first three volumes (5.7 s) of the functional data were discarded. A corrected cluster significance threshold of *p* < 0.05 was applied to the *Z*‐statistic images after thresholding at *Z* > 3.1.

In higher level analysis, separate mixed effects regression models were performed for each of the independent variables (MV‐PA, sedentary time, overall appetite, appetite‐related hormones and glucose) to explore associations with the food‐cue‐related contrasts generated at the first level (i.e., the dependent variable). Model 1 included age, sex and device weartime (for behavioural variables) as covariates, and model 2 was further adjusted for BMI. Significant associations for behavioural exposures (MV‐PA, sedentary time) in model 2 were investigated further in exploratory analyses by adding sequentially an interaction term for sex, age and BMI to assess whether the associations were modified by these variables. In a sensitivity analysis (model 3), we explored whether the associations for behavioural exposures (MV‐PA, sedentary time) were independent of each other. Inactivity obtained from the wrist‐worn monitor was used for this model instead of sedentary time from the thigh‐worn monitor to ensure the movement behaviours were captured on identical days by the same measuring device. Model 3 for MV‐PA was adjusted for the same covariates as Model 2 plus inactivity obtained from the wrist‐worn monitor, and a separate model was constructed with inactivity from the wrist‐worn monitor as the dependent variable, with adjustment for age, sex, BMI, device weartime and MV‐PA. Multicollinearity was assessed between MV‐PA and inactivity in model 3 using the variance inflation factor, with values in all models ≤ 2.0 indicating that multicollinearity was low. The Harvard‐Oxford cortical and subcortical atlases were specified in the atlas query tool in FSL (and the associated autoaq script) to obtain information about the peak value of activated clusters, with data reported for clusters containing ≥ 10 continuous voxels.

### 2.11 Regions of Interest Analysis

In line with our previous work (Thackray et al. 2023), seven pre‐identified regions of interest (ROIs) were examined, which have been implicated in the processing of visual food cues: amygdala, hippocampus, hypothalamus, insula, nucleus accumbens, OFC and striatum (Huerta et al. 2014; Killgore et al. 2003; Tang et al. 2012; van der Laan et al. 2011; van Meer et al. 2015). Associations between the independent variables and food cue reactivity (food (HED + LED) vs. non‐food, HED vs. non‐food and LED vs. non‐food) in each ROI mask were examined in FSL's Randomise with adjustment for age, sex, BMI and device weartime (behavioural variables only) (model 2), with further exploratory analyses performed for behavioural exposures (MV‐PA, sedentary time) by adding an interaction term for sex, age and BMI. Additional sensitivity models were performed for behavioural variables that included mutual adjustment for MV‐PA and inactivity from the wrist‐worn monitor (model 3). A threshold‐free cluster enhancement (TFCE) and a family‐wise corrected P value of 0.05 were applied, and a Bonferroni adjustment was used to account for multiple ROI comparisons. FSL's cluster command was used to identify the peak of activated clusters, and clusters containing at least 10 continuous voxels are reported.

### 2.12 Sample Size Justification

A pragmatic approach was taken for the sample recruited to the study due to resource constraints and the absence of previous data on device‐measured physical activity and brain food cue responsivity (Lakens 2022). Using the *pwr* package for general linear models in R (version 4.2.2), a moderate Cohen's *f* ^2^ of 0.29 could be detected with a target sample size of 50, assuming an alpha of 0.05 and 80% power in our fully adjusted model. The 95% confidence interval of the target *f* ^2^ of 0.29 was calculated as 0.05 to 0.68 for a sample size of 50 participants and five predictors.

## 3 Results

### 3.1 Participants and Missing Data

This study recruited 51 healthy adults (30 men, 21 women) with the physical and physiological characteristics of participants presented in Table 1. Data for MV‐PA is presented for 50 participants as one participant did not meet the minimum device weartime criteria. Forty‐four participants were included for sedentary time due to technical issues with the device during data collection (*n* = 5) or non‐compliance with the minimum device weartime criteria (*n* = 2). Biochemical outcomes are presented for 48 participants due to missing venous blood samples.

**TABLE 1:** Participant characteristics.

Variable	Combined (*n* = 51)		Men (*n* = 30)	Women (*n* = 21)
	Mean ± SD	Range (min–max)	Mean ± SD	Mean ± SD
Age (years)	26 ± 6	18 to 44	26 ± 6	26 ± 7
Handedness (*n* right/*n* left)	47/4		27/3	20/1
Stature (m)	1.73 ± 0.10	1.54 to 1.89	1.80 ± 0.07	1.64 ± 0.06
Body mass (kg)	73.0 ± 14.6	50.1 to 112.0	81.8 ± 11.8	60.3 ± 6.2
Body mass index (kg/m^2^)	24.1 ± 3.0	18.5 to 32.7	25.3 ± 2.8	22.4 ± 2.2
Waist circumference (cm)	81.3 ± 10.9	64.0 to 100.0	87.5 ± 7.7	72.5 ± 8.7
Body fat percentage (%)	24.2 ± 7.7	3.7 to 39.7	21.3 ± 7.8	28.5 ± 5.3
Cognitive restraint (TFEQ)	8 ± 4	1 to 18	8 ± 4	8 ± 4
Disinhibition (TFEQ)	6 ± 3	2 to 14	6 ± 3	6 ± 3
Hunger (TFEQ)	5 ± 3	0 to 12	6 ± 3	5 ± 3
MV‐PA (minutes/day)	77 ± 37	20 to 208	74 ± 37	82 ± 36
Sedentary time (minutes/day)	578 ± 83	388 to 808	605 ± 89	543 ± 59
Total GLP‐1 (pmol/L)	42.1 ± 14.5	15.4 to 78.1	44.3 ± 14.0	38.7 ± 15.1
Total PYY (pg/mL)	147 ± 37	62 to 273	142 ± 35	153 ± 41
Acylated ghrelin (pg/mL)	258 ± 298	30 to 1723	199 ± 244	348 ± 353
Glucose (mmol/L)	4.88 ± 0.37	4.21 to 6.10	4.93 ± 0.38	4.81 ± 0.36
Overall appetite (mm)	65 ± 19	22 to 97	68 ± 19	61 ± 18

*Note:* Data presented for *n* = 51 apart from MV‐PA (*n* = 50), sedentary time (*n* = 44) and biochemical outcomes (*n* = 48).

Abbreviations: GLP‐1, glucagon‐like peptide 1; MV‐PA, moderate‐to‐vigorous intensity physical activity; PYY, peptide tyrosine tyrosine; TFEQ, three factor eating questionnaire.

### 3.2 BOLD Associations With Behavioural Outcomes

**FIGURE 2:**
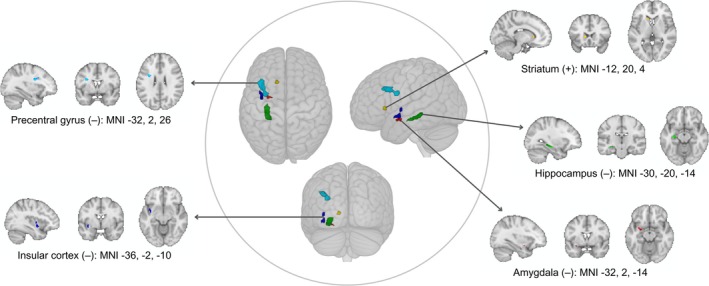
Association between device‐measured moderate‐to‐vigorous intensity physical activity (MV‐PA) and the blood‐oxygen‐level‐dependent (BOLD) signal change in response to LED versus non‐food cues (*n* = 50 men and women). Clusters of activation were identified in analysis performed at the whole‐brain level (precentral gyrus) and in pre‐specified regions of interest (hippocampus, insula, amygdala, striatum). The direction of association between MV‐PA and BOLD food cue reactivity is denoted in brackets: (+) positive or (−) negative. Models were adjusted for age, sex, body mass index and device weartime. Brain maps presented in neurological convention with the left hemisphere shown on the left. LED, very low‐ and low‐energy density foods; MNI, Montreal Neurological Institute.

**FIGURE 3:**
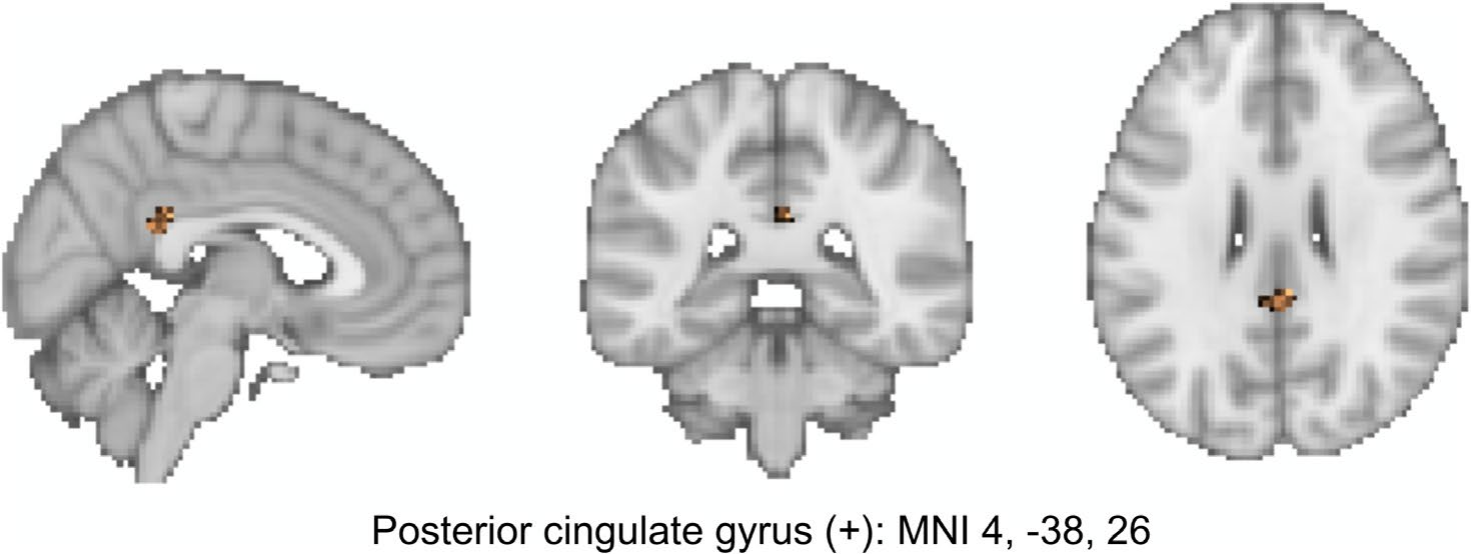
Positive association between device‐measured sedentary time and the blood‐oxygen‐level‐dependent (BOLD) signal change in response to HED versus non‐food cues (*n* = 44 men and women). Cluster identified in the whole‐brain analysis with adjustment for age, sex, body mass index and device weartime. Brain maps presented in neurological convention with the left hemisphere shown on the left. HED, high‐ and very high‐energy density foods; MNI, Montreal Neurological Institute.

Associations of MV‐PA and sedentary time with brain food cue responsiveness are presented in Table 2. At the whole brain level, MV‐PA was negatively associated with reactivity to LED versus non‐food cues in the middle frontal gyrus (model 1) and the precentral gyrus (model 2; Figure 2). After adjustment for age, sex, device weartime (model 1) and BMI (model 2), a positive association was identified between sedentary time and BOLD activity in response to food (HED + LED) versus non‐food cues and HED versus non‐food cues in the posterior cingulate gyrus (Figure 3). Sedentary time was also positively associated with reactivity to LED versus non‐food cues in the paracingulate gyrus and negatively associated with reactivity to food (HED + LED) versus non‐food cues in the central opercular cortex in model 1, but these associations were no longer evident after further adjustment for BMI (model 2). Exploratory interaction analyses did not identify any moderating influence of sex, age or BMI on the associations identified in model 2.

**TABLE 2:** Whole brain and regions of interest analysis showing associations of device‐measured moderate‐to‐vigorous intensity physical activity (MV‐PA) and sedentary time with the blood‐oxygen‐level‐dependent signal change in response to visual food cues.

Model	Contrast	Direction	Brain region	Hemisphere	No. of voxels	MNI brain coordinates			*z* value
						*x*	*y*	*z*	
**MV‐PA**									
Model 1	Food (HED + LED) > non‐food	No activated clusters after correction for multiple comparisons							
	HED > non‐food	No activated clusters after correction for multiple comparisons							
	LED > non‐food	Negative	Middle frontal gyrus	Left	137	−36	22	34	4.18
Model 2	Food (HED + LED) > non‐food	Negative	Hippocampus*	Left	60	−30	−18	−14	4.69
	HED > non‐food	No activated clusters after correction for multiple comparisons							
	LED > non‐food	Negative	Precentral gyrus	Left	141	−32	2	26	4.60
		Negative	Hippocampus*	Left	99	−30	−20	−14	4.80
		Negative	Insular cortex (posterior)*	Left	43	−36	−2	−10	4.84
		Negative	Amygdala*	Left	21	−32	2	−14	4.43
		Positive	Striatum (caudate nucleus)*	Left	17	−12	20	4	5.04
**Sedentary time**									
Model 1	Food (HED + LED) > non‐food	Positive	Posterior cingulate gyrus	Right	85	2	−42	24	4.98
		Negative	Central opercular cortex	Right	81	38	0	14	4.16
	HED > non‐food	Positive	Posterior cingulate gyrus	Right	93	0	−42	24	4.18
	LED > non‐food	Positive	Paracingulate gyrus	Right	79	2	40	34	4.03
Model 2	Food (HED + LED) > non‐food	Positive	Posterior cingulate gyrus	Right	82	2	−42	24	5.20
	HED > non‐food	Positive	Posterior cingulate gyrus	Right	90	4	−38	26	4.24
	LED > non‐food	No activated clusters after correction for multiple comparisons							

*Note:* Results represent the direction of association, brain region identified from Harvard‐Oxford cortical or subcortical probabilistic atlases, right or left brain hemisphere, the number of voxels in each cluster (2.2 mm^3^; minimum cluster size of 10 voxels), and the coordinates in MNI space and *z* value for the peak statistical voxel.

Abbreviations: HED, high‐ and very high‐energy density foods; LED, very low‐ and low‐energy density foods; MNI, Montreal Neurological Institute.

* Clusters detected in the regions of interest (ROI) analysis; all other clusters identified in the whole‐brain analysis (MV‐PA *n* = 50, sedentary time *n* = 44). Whole‐brain group‐level statistical analysis was performed using a higher‐level mixed effects model in FMRIB's Expert Analysis Tool (FEAT). The *z*‐statistic image for each contrast was thresholded at *z* > 3.1 using a corrected cluster significance threshold of *p* < 0.05. Regions of interest analysis was performed using a non‐parametric permutation approach in Randomise, applying threshold‐free cluster enhancement (TFCE), a family‐wise error corrected *p* value of *p* < 0.05, and a Bonferroni correction for multiple ROI comparisons. Model 1 includes adjustment for age, sex, and device weartime. Model 2 includes the same covariates as model 1, with further adjustment for BMI.

In the ROI analysis, fully adjusted models revealed a negative association between MV‐PA and BOLD activity in response to food (HED + LED) versus non‐food cues (*p* = 0.010) and LED versus non‐food cues (*p* = 0.004) in the hippocampus (Figure 2). Furthermore, in the LED versus non‐food contrast, MV‐PA was negatively associated with food cue reactivity in the insular cortex (posterior) (*p* = 0.011) and amygdala (*p* = 0.027) and positively associated with food cue reactivity in the dorsal striatum (caudate nucleus) (*p* = 0.024) (Figure 2). No BOLD‐behaviour relationships were identified in any of the ROIs for sedentary time. The associations identified in the ROI analysis for MV‐PA were not moderated by sex, age or BMI in exploratory interaction analysis.

#### 3.2.1 Sensitivity Analysis (Model 3)

Sensitivity models for MV‐PA with additional adjustment for inactivity from the wrist‐worn device revealed no BOLD‐behaviour associations in the whole‐brain analysis, but several negative associations were identified in the ROI analysis that were generally consistent with the findings from model 2 (Table S1, Figure S1). Analysis of the inactivity data from the wrist‐worn device with adjustment for age, sex, BMI, device weartime and MV‐PA revealed no clusters of activation in either the whole brain or ROI models.

### 3.3 BOLD Associations With Appetite‐Related and Metabolic Outcomes

Associations of appetite‐related and metabolic outcomes with brain food cue responsiveness are presented in Table 3. At the whole brain level, after adjustment for age and sex (model 1), fasting total GLP‐1 was negatively associated with reactivity to food (HED + LED) versus non‐food cues in the anterior supramarginal gyrus, putamen, frontal pole, insular cortex (posterior), precentral gyrus and middle frontal gyrus. These clusters of activation persisted after additional adjustment for BMI (model 2) apart from the frontal pole. In the LED versus non‐food contrast, a negative association was identified between fasting total GLP‐1 and BOLD activity in the putamen, postcentral gyrus, precentral gyrus, frontal pole and lateral occipital cortex (models 1 and 2). Additional clusters of activation demonstrating negative associations with fasting total GLP‐1 were also identified in the insular cortex (dorsal anterior) and anterior supramarginal gyrus of fully adjusted models (model 2).

**TABLE 3:** Whole brain and regions of interest analysis showing associations of plasma total peptide YY (PYY), total glucagon‐like peptide‐1 (GLP‐1) and glucose with the blood‐oxygen‐level‐dependent signal change in response to visual food cues.

Model	Contrast	Direction	Brain region	Hemisphere	No. of voxels	MNI brain coordinates			*z* value
						*x*	*y*	*z*	
**Total GLP‐1**									
Model 1	Food (HED + LED) > non‐food	Negative	Anterior supramarginal gyrus	Right	349	64	−20	40	4.20
		Negative	Anterior supramarginal gyrus	Left	228	−66	−24	24	3.98
		Negative	Putamen	Left	133	−20	10	−12	4.05
		Negative	Frontal pole	Right	115	50	38	8	4.20
		Negative	Insular cortex (posterior)	Right	103	40	2	−14	4.46
		Negative	Precentral gyrus	Left	90	−58	8	6	4.29
		Negative	Middle frontal gyrus	Left	82	−44	34	20	4.59
	HED > non‐food	No activated clusters after correction for multiple comparisons							
	LED > non‐food	Negative	Putamen	Right	457	28	8	−8	4.95
		Negative	Postcentral gyrus	Right	416	60	−18	36	4.47
		Negative	Putamen	Left	406	−22	10	−6	4.91
		Negative	Postcentral gyrus	Left	251	−60	−22	26	4.06
		Negative	Precentral gyrus	Left	227	−58	8	6	4.25
		Negative	Frontal pole	Right	154	52	38	8	4.06
		Negative	Precentral gyrus	Right	119	52	8	22	4.45
		Negative	Frontal operculum cortex	Right	108	36	18	6	4.03
		Negative	Lateral occipital cortex	Right	93	38	−80	30	3.85
		Negative	Intracalcarine cortex	Right	83	14	−74	8	3.95
Model 2	Food (HED + LED) > non‐food	Negative	Anterior supramarginal gyrus	Right	327	64	−30	34	4.25
		Negative	Anterior supramarginal gyrus	Left	261	−54	−26	34	4.16
		Negative	Putamen	Left	161	−22	6	−2	4.13
		Negative	Insular cortex (posterior)	Right	133	40	6	−14	4.50
		Negative	Striatum (putamen)*	Left	104	−22	10	−10	4.33
		Negative	Precentral gyrus	Left	85	−60	8	6	4.34
		Negative	Middle frontal gyrus	Left	80	−44	34	18	4.53
		Negative	Striatum (putamen)*	Right	32	18	10	−8	4.19
	HED > non‐food	No activated clusters after correction for multiple comparisons							
	LED > non‐food	Negative	Putamen	Right	443	28	8	−8	5.23
		Negative	Putamen	Left	408	−22	10	−6	4.82
		Negative	Postcentral gyrus	Right	395	60	−18	36	4.42
		Negative	Anterior supramarginal gyrus	Left	295	−60	−24	26	4.18
		Negative	Precentral gyrus	Left	206	−54	4	16	4.18
		Negative	Striatum (putamen)*	Left	143	−22	12	−10	4.91
		Negative	Frontal pole	Right	142	52	38	8	4.01
		Negative	Insular cortex (dorsal anterior)*	Right	110	40	12	2	5.06
		Negative	Insular cortex (dorsal anterior)	Right	98	36	16	2	4.04
		Negative	Precentral gyrus	Right	96	52	8	22	4.36
		Negative	Striatum (putamen)*	Right	91	18	10	−8	5.04
		Negative	Lateral occipital cortex	Right	85	34	−78	36	3.86
		Negative	Striatum (putamen)*	Right	55	30	6	−2	5.07
		Negative	Amygdala*	Right	15	16	0	−16	4.16
		Negative	Striatum (putamen)*	Right	11	28	−10	10	5.11
**Total PYY**									
Model 1	Food (HED + LED) > non‐food	Negative	Middle frontal gyrus	Right	100	28	14	54	4.49
	HED > non‐food	Negative	Middle frontal gyrus	Right	136	30	14	56	4.57
	LED > non‐food	No activated clusters after correction for multiple comparisons							
Model 2	Food (HED + LED) > non‐food	Negative	Middle frontal gyrus	Right	97	30	14	54	4.52
	HED > non‐food	Negative	Middle frontal gyrus	Right	123	30	14	56	4.66
		Negative	Precentral gyrus	Left	86	−24	−12	52	3.99
	LED > non‐food	No activated clusters after correction for multiple comparisons							
**Glucose**									
Model 1	Food (HED + LED) > non‐food	Negative	Postcentral gyrus	Left	81	−44	−12	26	4.98
	HED > non‐food	No activated clusters after correction for multiple comparisons							
	LED > non‐food	No activated clusters after correction for multiple comparisons							
Model 2	Food (HED + LED) > non‐food	No activated clusters after correction for multiple comparisons							
	HED > non‐food	No activated clusters after correction for multiple comparisons							
	LED > non‐food	No activated clusters after correction for multiple comparisons							

*Note:* Results represent the direction of association, brain region identified from Harvard‐Oxford cortical or subcortical probabilistic atlases, right or left brain hemisphere, the number of voxels in each cluster (2.2 mm^3^; minimum cluster size of 10 voxels), and the coordinates in MNI space and *z* value for the peak statistical voxel.

Abbreviations: HED, high‐ and very high‐energy density foods; LED, very low‐ and low‐energy density foods; MNI, Montreal Neurological Institute.

* Clusters detected in the regions of interest (ROI) analysis; all other clusters identified in the whole‐brain analysis (*n* = 48 participants). Whole‐brain group‐level statistical analysis was performed using a higher‐level mixed effects model in FMRIB's Expert Analysis Tool (FEAT). The *z*‐statistic image for each contrast was thresholded at *z* > 3.1 using a corrected cluster significance threshold of *p* < 0.05. Regions of interest analysis was performed using a non‐parametric permutation approach in Randomise, applying threshold‐free cluster enhancement (TFCE), a family‐wise error corrected P value of *p* < 0.05, and a Bonferroni correction for multiple ROI comparisons. Model 1 includes adjustment for age and sex. Model 2 includes the same covariates as model 1 with further adjustment for BMI.

In the whole brain analysis, fasting total PYY was negatively associated with the BOLD signal change in the middle frontal gyrus in response to food (HED + LED) versus non‐food cues and HED versus non‐food cues (models 1 and 2). A negative association was also identified for fasting total PYY in the fully adjusted model (model 2) of the HED versus non‐food contrast in the precentral gyrus. Fasting glucose was negatively associated with reactivity to food (HED + LED) versus non‐food cues in the postcentral gyrus (model 1), but this relationship did not persist after further adjustment for BMI (model 2). Fasting acylated ghrelin and overall appetite were not associated with food cue reactivity in any of the contrasts (models 1 and 2).

In the ROI analysis, fully adjusted models revealed a negative association between fasting total GLP‐1 and BOLD activity in response to food (HED + LED) versus non‐food cues in the dorsal striatum (putamen) (*p* = 0.007). In the LED versus non‐food contrast, fasting total GLP‐1 was negatively associated with the BOLD signal change in the dorsal striatum (putamen), insular cortex (dorsal anterior) and amygdala (all *p* ≤ 0.024). Fasting total PYY, acylated ghrelin, glucose and overall appetite were not associated with food cue reactivity in any of the ROIs.

## 4 Discussion

This study investigated the relationship of device‐measured physical activity and sedentary time with neural responses to visual food cues in healthy men and women. The key findings are as follows: (1) time spent in MV‐PA was inversely related to food cue reactivity in the precentral gyrus, hippocampus, posterior insula and amygdala; (2) a positive association was identified between MV‐PA and food cue reactivity in the dorsal striatum and (3) sedentary time was positively associated with food cue reactivity in the posterior cingulate gyrus. Importantly, these relationships were identified after adjusting for key demographic, adiposity and device‐related variables that could confound the associations, and were not moderated by sex, age or BMI.

In the present study, we identified novel inverse relationships between device‐measured MV‐PA and reactivity to food cues, particularly of low energy density, in the hippocampus, posterior insula and amygdala. These findings extend previous observations that self‐reported physical activity is negatively associated with the BOLD signal change in response to food versus non‐food cues in the insula (Killgore et al. 2013; Luo et al. 2018) and amygdala (Luo et al. 2018). The hippocampus, insula and amygdala are consistently activated in response to food stimuli (Huerta et al. 2014; Tang et al. 2012; van der Laan et al. 2011; van Meer et al. 2015) and harbour wide‐ranging roles in brain‐related eating behaviour (Azevedo et al. 2022; Berthoud et al. 2017).

Within this context, the posterior insula plays a crucial role in processing interoceptive signals, including feelings of hunger and fullness (Craig 2002) and has been associated with satiety (Morris and Dolan 2001). The hippocampus is primarily implicated in integrating information from internal and external cues with episodic meal‐related memories (Parent et al. 2022; Kanoski and Grill 2017), with the retrieval of meal‐related memories associated with lower subsequent food intake (Parent et al. 2022). A potential interpretation of our findings is that MV‐PA may enhance the appeal of foods low in energy density through lower activation in the satiety‐related division of the insula and by attenuating the inhibitory influence of meal‐related memories on eating healthier, lower energy value foods. In contrast, it is well established that the amygdala is involved in encoding the motivational and emotional value of a (food) stimulus (Gottfried et al. 2003), which implies that those engaging in higher levels of MV‐PA may assign lower hedonic value to lower energy density foods during the anticipation of food. Whilst this appears contradictory, these brain regions have complex and diverse roles (Berthoud et al. 2017), and the inference that time spent in MV‐PA is associated with enhanced appeal of and motivation to consume healthier foods appears consistent with the wider literature adopting non‐imaging techniques. Specifically, higher levels of physical activity have been associated with lower liking and wanting of high‐fat foods (Beaulieu et al. 2020) and enhanced postprandial satiety signaling (Beaulieu et al. 2017).

The contention that MV‐PA may lower inhibition towards LED foods is further supported by the inverse association identified in the whole brain analysis between device‐measured MV‐PA and the BOLD signal change in the middle frontal gyrus in response to LED versus non‐food cues. Indeed, this region of the frontal lobe is closely associated with inhibitory processing (Nakata et al. 2008) and stimulus‐oriented attention (Corbetta and Shulman 2002). This voxel cluster was identified in the model unadjusted for BMI, but after accounting for the potential confounding influence of BMI, a similar cluster of activation representative of an inverse association was identified in the precentral gyrus. Although the role of the precentral gyrus in brain food cue responsiveness is not well recognized, the close vicinity of the middle frontal and precentral gyri clusters in our analysis makes it unlikely that the underlying influence on eating behaviour within this data is markedly different. Our analysis also identified a positive association between device‐measured MV‐PA and reactivity to LED versus non‐food cues in the dorsal striatum, a region that is implicated in food motivation through dopaminergic projections (Volkow et al. 2002). Therefore, greater engagement in MV‐PA may enhance the motivation to consume LED foods. This is supported by previous work highlighting that food cue responsiveness in the nucleus accumbens, located on the reward pathway in the ventral striatum, is predictive of subsequent energy intake (Lawrence et al. 2012).

Another key finding to emerge from the current study was that device‐measured sedentary time was positively related to food cue reactivity in the posterior cingulate gyrus. This finding was apparent both with and without adjustment for BMI and appeared particularly pertinent in response to HED food images. The only previous investigation exploring relationships between sedentary time and brain food cue responsiveness identified positive associations in the mid insula and amygdala after controlling for age and obesity status (Luo et al. 2018). It is unclear why our analysis identified different brain–behaviour relationships, but the objective quantification of sedentary time in the present study is likely to capture this behaviour more precisely (Dhurandhar et al. 2015). Nevertheless, the voxel cluster identified in the posterior cingulate gyrus was situated primarily within the dorsal division that has been implicated in controlling attentional focus through monitoring and responding to environmental stimuli (Leech and Sharp 2014; Foster et al. 2023). Thus, the heightened reactivity in this region in those engaged in higher levels of sedentary time may reflect a greater allocation of attention towards HED food stimuli in anticipation of reward. The partially adjusted model for sedentary time also identified a negative association in the central opercular cortex (food vs. non‐food) and a positive association in the paracingulate gyrus (LED vs. non‐food). The relevance of these associations is unclear, and they were no longer apparent after additional adjustment for BMI. Considering BMI did not moderate the strength of any of the brain–behaviour relationships in the study, this could be because BMI is related to both sedentary time and food cue reactivity in these regions, so controlling for BMI weakened the association.

Gut‐derived peptides influence eating behaviour by interacting with homeostatic and reward‐related brain centres (Althubeati et al. 2022). The present study identified several brain sites demonstrating inverse associations between fasting total GLP‐1 concentrations and food cue reactivity. Most of these relationships persisted after controlling for BMI and included brain regions related to reward (putamen, anterior insula), emotional processing (amygdala, anterior supramarginal gyrus), attention (middle frontal gyrus, frontal pole) and interoceptive awareness (posterior insula). These findings appear consistent with previous investigations reporting that endogenous (Heni et al. 2015), exogenous (de Silva et al. 2011) and pharmacological (Farr et al. 2016) activation of the GLP‐1 system is associated with lower food cue‐induced brain activation within reward‐related regions such as the putamen, insula, amygdala and OFC. Furthermore, the inverse association identified between fasting total PYY concentrations and brain responses to food stimuli in the middle frontal gyrus appears in line with evidence showing that exogenous PYY infusion reduces the BOLD signal within this region of the frontal lobe (Batterham et al. 2007). However, the relevance of the associations identified for total GLP‐1 and total PYY in the present study is unclear given GLP‐1 and PYY circulate at low concentrations when fasted, and the total concentration integrates both the active and inactive isoforms of the hormones; but it is the biologically active form abundant in the postprandial state that provokes satiety effects in the brain (Batterham et al. 2007; de Silva et al. 2011; MacLean et al. 2017). Future investigations that quantify the different isoforms of the hormones in response to nutrient ingestion are required to provide a better insight into the brain regions modulated by satiety‐related peptides.

In fully adjusted models, we identified no associations between brain food cue responsiveness and fasting concentrations of acylated ghrelin and glucose or subjective appetite perceptions. This is somewhat surprising given that the infusion or injection of the orexigenic hormone ghrelin has been shown to activate multiple brain regions associated with reward, emotion and cognition, including the amygdala, OFC, anterior insula, hippocampus and striatum (Malik et al. 2008; Goldstone et al. 2014). Additionally, fasting ghrelin concentrations have been positively associated with food cue reactivity in visual processing and reward‐related brain regions, including those within the limbic system (Kroemer et al. 2013a; Wever et al. 2021). Although this inconsistency may reflect the form of ghrelin assessed (total: Wever et al. 2021; unacylated: Kroemer et al. 2013a), the measurement of the biologically active isoform in the present study might be expected to improve the sensitivity to detect meaningful relationships. The absence of relationships between fasting glucose concentrations and brain responses to food stimuli in our fully adjusted model has been replicated previously (Kroemer et al. 2013b) and likely reflects the low variance in glucose concentrations evident in our healthy sample (Table 1). Conversely, glucose ingestion appears to reduce the BOLD signal in homeostatic and hedonic‐related brain areas, which may contribute to lowering the rewarding value of food in the postprandial state (Kroemer et al. 2013b; Heni et al. 2014). In contrast with our findings, subjective appetite sensations collected immediately after a food cue paradigm have been linked to activation in the insula, operculum and putamen (Porubská et al. 2006). This disparity in findings may be attributable to differences in the method adopted to assess appetite. Specifically, we implemented validated visual analogue scales that capture the perceived general state of certain appetite constructs, whereas the aforementioned study assessed the motivational value of each food item presented during the food cue paradigm, which is likely to hold greater specificity to the preceding brain responsivity.

The strengths of our study include the use of accelerometry to objectively quantify free‐living physical activity and sedentary time in a heterogeneous sample of men and women. The food cue paradigm consisted of food items of known energy density that were divided into distinct low and high hedonic value categories to improve the ability to detect brain–behaviour relationships according to the energy value of food. Furthermore, physical activity and diet were carefully controlled in the 24 h before the lab‐based study assessments to mitigate any potential confounding influence. However, the observed relationships are limited by the cross‐sectional design that precludes the ability to establish causation and requires future longitudinal investigations to ascertain. The analysis was performed in a relatively small sample of young, physically active participants with, on average, a healthy BMI, and a loss of statistical power may have influenced some statistical models due to missing data in behavioural (MV‐PA, sedentary time) and biochemical outcomes. Consequently, the findings may not generalise to other populations such as older adults and individuals with obesity and/or impaired glycaemic control who may present different results and should be considered in future investigations. A myriad of factors play an integral role in shaping movement behaviours along the continuum from sleep to MV‐PA, as well as eating behaviours, including brain responses to food cues. These include sociocultural practices (e.g., eating habits, health attitudes), environmental influences (e.g., food availability, access to outdoor space), lifestyle components (e.g., work habits) and economic pressures, all of which could potentially influence the observed brain–behaviour relationships. Investigating the dynamic interplay of these components in diverse populations is needed to better understand the factors that moderate the association between movement behaviours and central appetite responses. Another limitation is that total GLP‐1 and PYY concentrations were measured rather than the biologically active forms (GLP‐1_7–36_ and PYY_3–36_, respectively). Finally, it is not possible to determine the direct relevance of the findings for feeding behaviour as energy intake was not assessed in conjunction with the other study measures.

In conclusion, this study identified associations between device‐measured MV‐PA and food cue reactivity, particularly when viewing low energy value foods, that were negative in direction within the precentral gyrus, hippocampus, posterior insula and amygdala, and positive in direction within the dorsal striatum. Device‐measured sedentary time was positively associated with food cue responsiveness in the posterior cingulate gyrus. These relationships lend support to the notion that MV‐PA may enhance the appeal of and motivation to consume foods of low energy density, whereas sedentary time may promote attention towards high‐energy value food stimuli. This suggests that higher levels of MV‐PA and less sedentary time have the potential to positively influence the central (brain) appetite control system. Additional longitudinal work in experimental settings is required to confirm whether the reported associations of MV‐PA and sedentary time with brain food cue responsiveness are causal.

## Conflicts of Interest

E.C.H. is employed by both the University of Bristol as a Senior Research Fellow (through which she completed this work) and on a part‐time basis with Oxford Medical Products as a Clinical Studies Manager. The work reported in this paper was conducted fully independently from the industry role. R.L.B. reports funding from the Sir Jule Thorn Trust, The Rosetrees Trust and the National Institute for Health and Social Care Research. R.L.B. reports receiving consulting fees from Pfizer, Eli‐Lilly, Gila Therapeutics Inc., and ViiV Healthcare and consulting fees, and lecture fees from Novo Nordisk, and participating in clinical trials for Novo Nordisk. From May 2023, she is a full‐time employee and shareholder for Eli‐Lilly and Company Ltd., Basingstoke, UK. The other authors declare no conflicts of interest.

## Supporting information


**Data S1.** Supporting Information.

## Data Availability

The de‐identified neuroimaging, physiological, and behavioural data generated in this study are available from the corresponding authors upon reasonable request.
