# Supplementary material for: Associations of Device‐Measured Physical Activity and Sedentary Time With Neural Responses to Visual Food Cues in Adults: A Functional Magnetic Resonance Imaging Study
Source: Hum Brain Mapp. 2025 Mar 12;46(4):e70192. doi: 10.1002/hbm.70192 (PMC11897806; doi:10.1002/hbm.70192)
Supplement: Supplementary file 1 — Data S1. Supporting Information. [file HBM-46-e70192-s001.pdf]

Supplementary material

Title: Associations of device-measured physical activity and sedentary time with neural responses to visual food cues in adults: a functional magnetic resonance imaging study

Authors: Abdulrahman M. Dera, Elanor C. Hinton, Rachel L. Batterham, Melanie J. Davies, James A. King, Masashi Miyashita, Paul S. Morgan, Dimitris Papamargaritis, Julie Thompson, David J. Stensel, Alice E. Thackray

Contents

Supplementary results

Supplementary Table 1 2
Supplementary Figure 1 3

Supplementary Table 1. Regions of interest sensitivity analysis showing associations between device-measured moderate-to-vigorous intensity physical activity (MV-PA) and the blood-oxygen-level-dependent signal change in response to visual food cues.

Model	Contrast	Direction	Brain region	Hemisphere	No. of voxels	MNI brain coordinates			*z* value
						x	y	z	
**MV-PA**									
Model 3	Food (HED + LED) > non-food	Negative	Insular cortex (posterior)	Left	36	-36	-4	-8	4.77
		Negative	Striatum (putamen)	Right	32	22	0	10	5.61
	HED > non-food	No activated clusters after correction for multiple comparisons							
	LED > non-food	Negative	Insular cortex (posterior)	Left	88	-40	6	-12	4.74
		Negative	Hippocampus	Left	36	-28	-36	-8	5.11
		Negative	Insular cortex (posterior)	Right	21	38	0	-12	5.35
		Negative	Amygdala	Right	17	30	0	-18	5.07
		Negative	Striatum (putamen)	Right	15	22	0	10	5.31

Regions of interest analysis performed using a non-parametric permutation approach in Randomise applying threshold-free cluster enhancement (TFCE), a family-wise error corrected P value of $P < 0.05$, and a Bonferroni correction for multiple ROI comparisons ($n = 50$). Model 3 includes adjustment for age, sex, BMI, device weartime and inactivity derived from the wrist-worn ActiGraph wGT3X-BT device.

Results represent the direction of association, brain region identified from Harvard-Oxford cortical or subcortical probabilistic atlases, right or left brain hemisphere, the number of voxels in each cluster (2.2 mm^3 ; minimum cluster size of 10 voxels), and the coordinates in MNI space and z value for the peak statistical voxel.

MNI, Montreal Neurological Institute; HED, high and very high-energy-density foods; LED, very low and low-energy density foods.

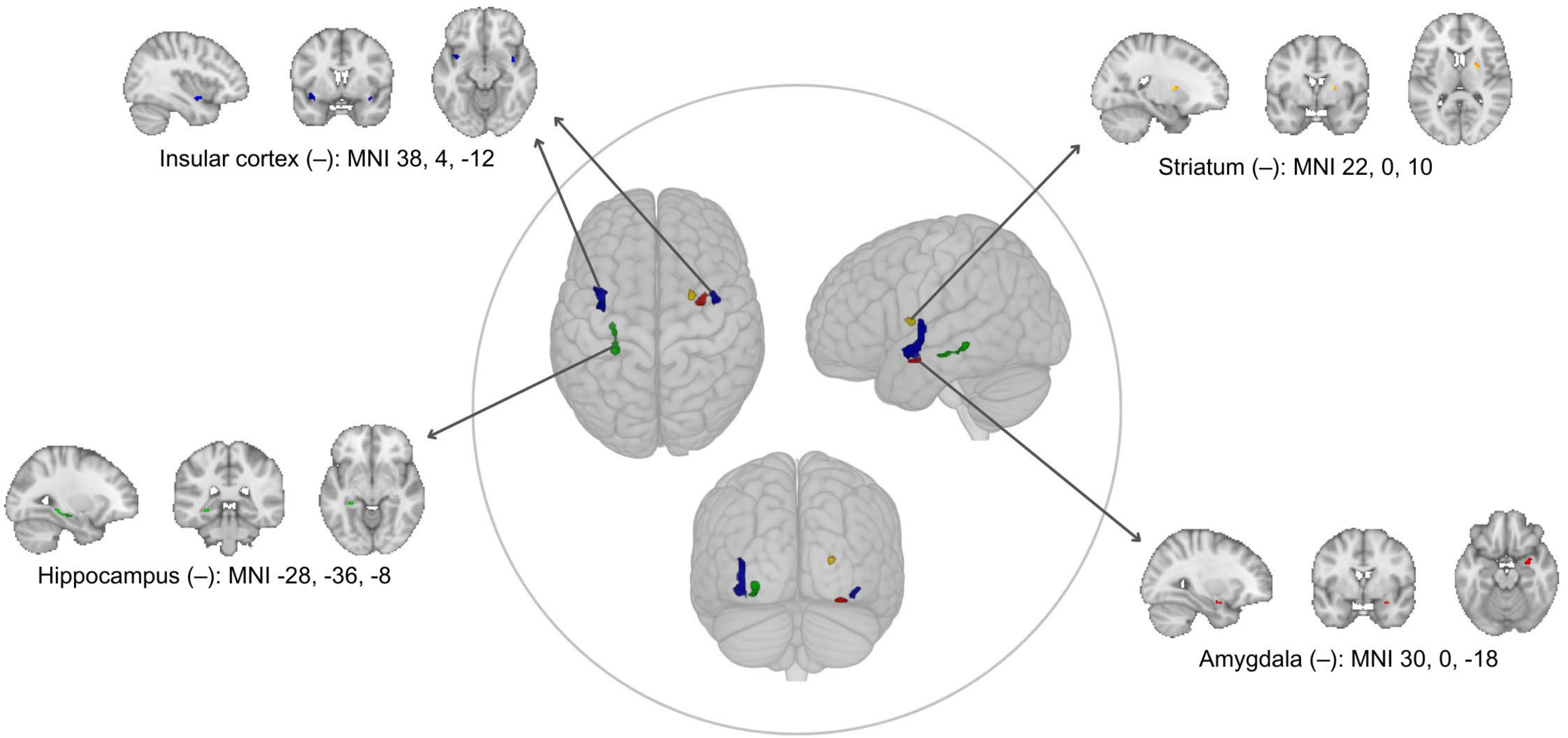

Supplementary Figure 1. Sensitivity analysis (model 3) showing negative (–) associations between device-measured moderate-to-vigorous intensity physical activity (MV-PA) and the blood-oxygen-level-dependent (BOLD) signal change in response to LED vs non-food cues (n = 50 men and women). Clusters of activation were identified in pre-specified regions of interest. Models were adjusted for age, sex, BMI, device

weartime and inactivity derived from the wrist-worn ActiGraph wGT3X-BT device. Brain maps presented in neurological convention with the left hemisphere shown on the left. LED, very low- and low-energy density foods; MNI, Montreal Neurological Institute.
